# Observation of the Therapeutic Effect of Washed Microbiota Transplantation on Childhood Autism Spectrum Disorder

**DOI:** 10.62641/aep.v54i2.2120

**Published:** 2026-04-15

**Authors:** Jingwen Li, Juan Liu, Mo Chen, Yi Wang, Cheng Zhou

**Affiliations:** ^1^Department of Pediatrics, The Second People’s Hospital of Changzhou, The Third Affiliated Hospital of Nanjing Medical University, 213003 Changzhou, Jiangsu, China; ^2^Microecology Center, The Second People’s Hospital of Changzhou, The Third Affiliated Hospital of Nanjing Medical University, 213003 Changzhou, Jiangsu, China; ^3^Chengdu High-Tech Zhiji Future Clinical Medical Laboratory, 610000 Chengdu, Sichuan, China; ^4^Department of Neurology, Children’s Hospital of Fudan University, 201102 Shanghai, China

**Keywords:** washed microbiota transplantation, intestinal flora, autism, efficacy, safety

## Abstract

**Background:**

This retrospective study evaluated the efficacy and safety of washed microbiota transplantation (WMT) via trans colonic endoscopic administration tube for children with autism spectrum disorder (ASD).

**Methods:**

The clinical data of 19 children with ASD treated between November 2021 and December 2023 were analysed. The data included scores on the Autism Behaviour Checklist (ABC), Childhood Autism Rating Scale (CARS) and PedsQL™ 3.0 Gastrointestinal Symptoms Scales (PedsQL-GI) before treatment and one and six months post-WMT, as well as faecal 16S rRNA sequencing results (vs. healthy controls).

**Results:**

ABC, CARS and PedsQL-GI scores improved significantly over time (all *p *< 0.001, large effect sizes). CARS and PedsQL-GI scores decreased notably at one and six months after treatment. ABC scores reduced significantly only at six months posttreatment. PedsQL-GI scores at six months posttreatment further declined relative to those atone month posttreatment, whereas ABC and CARS scores remained stable. Subgroup analysis showed greater score reductions in the high-score ASD and constipation subgroups than in other patients. Faecal microbiota analysis revealed structural differences between ASD and healthy children. WMT altered gut flora structure and increased beneficial bacteria (e.g.,* Faecalibacterium*).

**Conclusions:**

Preliminary findings suggest that WMT may improve gastrointestinal and core symptoms in children with ASD, especially those in high-score subgroups. Caution is needed given this study’s small sample size, and large prospective studies are required for validation.

## Introduction

Autism spectrum disorder (ASD) is a group of neurodevelopmental disorders characterised by impairments in social interactions, repetitive stereotyped behaviours and communication disorders. Epidemiological surveys have found that the prevalence of autism has been increasing annually, and the latest data released by the Centers for Disease Control and Prevention (CDC) in 2023 show that the prevalence of ASD has risen to 1/36 [1]. In China, ASD has a prevalence of approximately 1% of school-age children [2]. The aetiology of autism remains unclear and may be related to genetic and environmental factors [3]. Effective drugs for treating the core symptoms of ASD in children do not exist.

Gut microbes form a complex bidirectional communication system between the gut and central nervous systems through immune, metabolic and neural pathways; this system is known as the microbe–brain–gut axis. The microbial–brain–gut axis plays an important role in brain development, immunity and metabolic homeostasis [4]. Dysregulated gut flora affects brain function through the neuroendocrine, neuroimmune and autonomic nervous systems; such an effect may lead to the development of neurodevelopmental disorders [5, 6]. A growing number of studies have found that the intestinal flora of patients with ASD differs from that of neurotypical individuals [7, 8]. Sterile mouse models have also demonstrated that microorganisms in the gut and their metabolites can influence autistic behaviour through the gut–brain axis [9]. Sterile mice transplanted with the gut flora from patients with autism exhibited behavioural features of autism. By contrast, the gut flora and its metabolites from normal healthy populations markedly improved behavioural abnormalities and modulated neural excitability in a mouse model of autism. This finding suggests that the intestinal flora can regulate mouse behaviour through the production of neuroactive metabolites, indicating that the microbe–gut–brain axis may play a key role in the development of ASD. The gut flora regulates the brain through multiple pathways [10], which can not only influence intestinal immune homeostasis but also regulate the development, maturation and function of microglia. In the endocrine pathway, the gut flora can regulate the secretion of neuropeptides. For example, it affects the secretion of serotonin. Certain gut microbes and their metabolites can interact directly with the enteric nervous system and vagus and spinal afferent nerves, generating local signals that can be involved in the regulation of cognition, mood and anxiety [11]. Related studies have found that probiotic supplementation improves gut dysbiosis and neurotransmitter disorders in children with ASD and can improve social behaviours in mice with ASD [12, 13]. Therefore, the approach of improving core symptoms of ASD by modulating gut microbes may be a potential target for the treatment of ASD [14]. While foundational studies have set the stage for understanding the microbiome’s role in ASD, randomised controlled trials in clinical settings have begun to explore practical applications. These studies have demonstrated the potential of probiotic interventions to ameliorate gut dysbiosis and neurotransmitter imbalances in children with ASD, suggesting a promising avenue for symptom management. However, existing research is not without its shortcomings. Variability in study design, sample size and microbial intervention specificity have led to inconsistent results. Additionally, the mechanistic understanding of how the gut flora influences ASD symptoms remains incomplete, with many pathways yet to be fully elucidated.

In consideration of the link between the gut and brain, faecal microbiota transplant (FMT) could be a viable therapeutic option for ASD. FMT refers to injecting various intestinal microorganisms, metabolites and natural antimicrobial substances isolated from the faeces of healthy people (donors) into the intestinal tract of patients through various methods (nasogastric intubation, duodenal intubation, gastroscopy and colonoscopy) to improve intestinal microecology and thus treat diseases caused by intestinal flora dysbiosis. FMT is currently the most effective treatment for recurrent *Clostridioides difficile* infection and has shown good therapeutic effects in the treatment of gastrointestinal diseases, such as inflammatory bowel disease and functional bowel disease [15]. Therefore, the treatment of ASD through the reconstruction of the intestinal flora, especially for patients with ASD and gastrointestinal symptoms [16], has received increasing attention. Washed microbiota transplantation (WMT) is based on traditional FMT, where the microbiota is washed repeatedly to remove harmful substances, thereby enhancing therapeutic effect and minimising adverse effects. Although preliminary research suggests the potential of WMT in managing ASD, the evidence base remains limited. This study aims to establish the safety and efficacy of WMT for ASD and elucidate the mechanistic roles of specific microbial strains in symptom modulation. Achieving these objectives will facilitate the optimisation of therapeutic protocols, enable personalised diagnostic and treatment strategies and ultimately improve the quality of life for individuals with autism.

## Materials and Methods

### Study Subjects and Data Collection

This study is a retrospective analysis. By reviewing the medical record system, 19 children with ASD who were diagnosed and received WMT treatment between November 2021 and December 2023 were included as study subjects. All the clinical data, scale assessment results and faecal sample test data of the children were extracted and analysed from historical records. All children met the diagnostic criteria for ASD in accordance with the Diagnostic and Statistical Manual of Mental Disorders, Fifth Edition [17]. The guardians of the children signed informed consent forms before treatment. The exclusion criteria were as follows: (1) recent infections; (2) severe malnutrition or immunodeficiency; (3) treatment with probiotics, antibiotics, or proton pump inhibitors within the past month; and (4) other generalised developmental and neurological disorders. This study was approved by the Clinical Medical Technology Ethics Committee of the Second People’s Hospital of Changzhou (approval no. [2024]YLJSC119).

### General Information

Amongst the 19 included children, 16 were male and three were female. Their ages ranged from 3 years to 18 years, with 10 cases aged 3–5 years, five cases aged 6–10 years, two cases aged 11–15 years and twocases aged 16–18 years. Additionally, 57.89% of the children exhibited symptoms of picky eating and food selectivity, 42.11% presented with constipation and 15.79% had sleep disturbances.

Data on scores on the Autism Behaviour Checklist (ABC), Childhood Autism Rating Scale (CARS) and PedsQL™ 3.0 Gastrointestinal Symptoms Scales (PedsQL-GI) were retrospectively collected for all children with ASD before transplantation [18, 19, 20], as well as at one month and six months after transplantation. The ABC scale consists of 57 items and yields a total score of 158. For the purpose of behavioural assessment in this study, a cut-off value of ≥67 points was implemented. The CARS scale has a total score of 60, with score ≥30 indicating a confirmed diagnosis of autism. The PedsQL-GI includes 10 core symptom dimensions related to gastrointestinal function (e.g., Stomach pain and hurt, heartburn and reflux, constipation and diarrhoea) with items scored on a 0–4 Likert scale (0 = never, one = almost never, 2 = sometimes, 3 = often, 4 = almost always), where high scores indicate severe gastrointestinal symptoms. This scale was used to quantify the baseline and postintervention gastrointestinal symptom burden of children.

In the pretransplantation baseline assessment of the 19 children, ABC scores were ≥67 in eight cases, 53–67 in seven cases and <53 in four cases, and CARS scores were ≥30 in 12 cases and <30 in seven cases. On the basis of baseline scores, children with ABC score ≥67 or CARS score ≥30 were classified into the high-severity subgroup (n = 12), whereas those with ABC score <67 and CARS score <30 were classified into the low-severity subgroup (n = 7). In terms of PedsQL-GI scores, five cases had scores <15, seven cases had scores between 15–30 and seven cases had scores >30.

### Historical Data on Donor Screening and Bacterial Fluid Preparation

Donors were sourced from the Microecology Centre of the Second People’s Hospital of Changzhou. The screening criteria for donors were as follows: (1) No family history of diabetes, rheumatoid immune disease and haematological oncological disease. (2) No history of infectious diseases and underwent serological examination to exclude hepatitis viruses, human immunodeficiency virus, cytomegalovirus, EBV, Mycobacterium tuberculosis, syphilis and TORCH viruses and Helicobacter pylori infections and faecal tests to exclude the presence of bacterial, viral, fungal and parasitic infections. (3) No use of antibiotics or microecological preparations within the last six months. (4) No psychopathology or mental health problems in parents or siblings within the family. (5) Evidence of typical living conditions, consumption of a regular diet and regular bowel movements.

Bacterial liquid was prepared by using the following protocol: Faeces collected from donors were processed into a homogeneous faecal suspension with saline. The resulting faecal suspension was then filtered by using a microfibre and a faecal preparation instrument. It was then centrifuged at 1100 ×g to obtain the bacterial precipitate. The precipitate was further centrifuged and washed three times with saline. Finally, 100 mL of saline was added to resuspend the bacterial precipitate. Each 40 mL of the washed bacterial solution contained approximately 1 × 10^13^ CFU viable bacteria.

### Review of Transplantation Methods

Data regarding all interventions and procedures for the included children were extracted and reviewed from the medical record system. All children underwent transendoscopic enteral tubing (TET) with the transanal injection of bacterial fluid. Seventeen children received two WMTs, whereas two children received one WMT. The children were evaluated by using the ABC, CARS and PedsQL-GI, and stool specimens were collected before transplantation and one month after transplantation. Forty-eight hours before tube placement, the children were put on a liquid diet, and two faecal specimens were collected. Fourteen hours prior to tube placement, the patient began fasting and taking oral polyethylene glycol 4000 for bowel preparation. Additionally, saline enemas were administered 1–2 times, depending on the level of bowel cleansing needed. Successful bowel cleansing was indicated by clear watery stool and no faecal residue. After completing the bowel preparation, the patient was taken to the endoscopy room to have a colonic indwelling TET tube inserted. Two hours after tube placement, the patient was fed with fluids. At 24, 48 and 72 h after tube placement, the patient received injections of 50 mL (<7 years old)/80 mL (≥7 years old) of washed bacterial solution [21]. This step was performed three times. The second transplantation was performed one month after the initial transplantation in a manner similar to that described above.

### Gut Microbiota Data Analysis

16S rRNA sequencing data from faecal samples collected from the children before and one month after transplantation were retrospectively analysed. This testing was completed by the Know Your Future Clinical Medical Laboratory. Simultaneously, sequencing data from the faecal samples of 18 healthy children of the same age retained in the authors’ institution during the same period were retrospectively included as an external reference. The data of the healthy control group were sourced from the authors’ institution’s established healthy children faecal sample bank. The children had no other significant neurological, gastrointestinal, or systemic diseases and served as a reference for general gut microbiota composition. Data analysis included raw data filtering, denoising, splicing and chimera removal to ensure the use of high-quality data for feature generation. This step was followed by species annotation, diversity analysis and community function prediction. The data were effectively grouped, and the differences between groups were compared and tested.

### Study Groups and Data Analysis Strategy

Multiple analysis sets were defined from the overall cohort to address the specific aims of this study. Their definitions and purposes are as follows:

The full analysis set (FAS) comprised all children who received at least one WMT session and one posttreatment assessment. This set was used for the primary analysis of intervention efficacy in the overall population.

The constipation subgroup of the FAS was defined by meeting the Rome IV diagnostic criteria for functional constipation at baseline. This subgroup was analysed to assess WMT efficacy in children with comorbid constipation.

Symptom severity groups were established as follows: Patients with scores meeting or exceeding the cut-offs (ABC ≥67 and CARS ≥30) were defined as the high-score group, whereas those with scores below the cut-offs were classified into the low-score group. This grouping strategy was designed to explore the potential association between baseline score characteristics and subsequent WM therapeutic efficacy. The selection of these cut-off values was based on the unique clinical context of the study population: all enrolled children had received prior systematic interventions in special schools, leading to generally reduced pre-treatment baseline scores that did not align with standard ASD diagnostic cut-offs for treatment-naive individuals. These groups were compared for baseline demographic and clinical characteristics.

The microbiome analysis cohort was a subset of the FAS who provided valid faecal samples at both predefined time points (pre-WMT group, post-first-WMT group and post-second-WMT group). This cohort was used for the longitudinal analysis of gut microbiota changes.

Treatment response groups were established as follows: On the basis of the postintervention Clinical Global Impression-Improvement (CGI-I) score [22], participants were classified as responders (CGI-I score of1 or 2) or non-responders (CGI-I score ≥3). This grouping was used to compare baseline gut microbiota profiles to identify potential predictive biomarkers.

### Statistical Methods

All statistical analyses were performed by using IBM SPSS Statistics for Windows (version 22.0), developed by IBM Corporation, located in Armonk, New York, the United States. The normality of continuous variables was verified by employing the Shapiro–Wilk test. All measurement data were confirmed to follow a normal distribution and were therefore expressed as mean ± standard deviation ($\bar{x}$ ± s). For intergroup comparisons of continuous variables, independent-samples *t*-test was used when variances were homogeneous. Paired *t*-test was applied for before-and-after comparisons within the same group. For analysing the main effect of time on scores across three time points (before treatment, one month posttreatment and six months posttreatment), repeated-measures analysis of variance (ANOVA) was utilised. Mauchly’s test of sphericity was performed to verify the sphericity assumption; the Greenhouse–Geisser correction was applied when the assumption was violated.

Hierarchical Bonferroni correction was applied with the following adjusted significance levels to control type I errors in multiple comparisons across the ABC, CARS and PedsQL-GI: (1) (α’) = 0.0167 (0.05/3) for the main time effect of each scale and (2) (α”) = 0.0056 (0.0167/3) for pairwise comparisons within each scale. Effect sizes were evaluated by using partial eta-squared (partial η^2^, ≥0.14 for large effect) for the overall main time effect and Cohen’s d (≥0.8 for large effect) for pairwise comparisons.

Categorical data were described as frequencies (percentages) and compared by using the chi-squared test. Fisher’s exact test was adopted when the expected frequency of any cell in the contingency table was <5 or the total sample size was small (n < 40). A two-tailed *p* value < 0.05 was considered statistically significant.

## Results

### Baseline Characteristics of the Study Cohort

No statistically significant difference in demographic characteristics were found between the low-score (n = 7) and high-score groups (n = 12) of the 19 children with ASD. The baseline demographic characteristics of the overall cohort and two subgroups are summarised in Table 1. Specifically, sex distribution (*p *= 1.000) and mean age (*p *= 0.430) were comparable. This result indicates that the two groups were well balanced at baseline, enhancing the validity of subsequent comparisons.

**Table 1.** **Baseline demographic characteristics and group comparisons of WMT patients**.

Characteristic	Overall (n = 19)	Low-score group (n = 7)	High-score group (n = 12)	*p *value
Male, n (%)	16 (84.21)	6 (85.71)	10 (83.33)	1.000
Age (years), Mean ± SD	7.37 ± 4.53	5.86 ± 2.34	8.25 ± 5.31	0.430

WMT, washed microbiota transplantation; SD, Standard Deviation.

### Clinical Symptom Assessment Results

#### ABC Scores

**Fig. 1.**
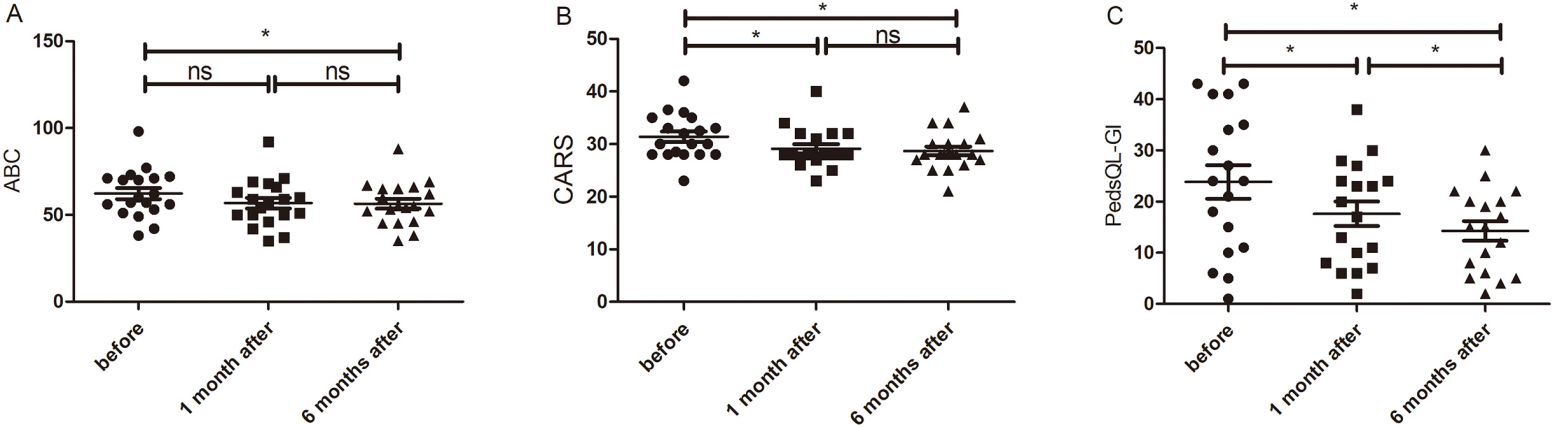
**Comparison of core symptom scores before and after treatment**. (A) Comparison of the ABC scores of children with ASD before treatment vs. those one month and six months posttreatment. (B) Comparison of CARS scores at pre-treatment and one month and six months posttreatment. (C) Comparison of PedsQL-GI scores at pre-treatment and one month and six months posttreatment. The “ns” indicates that the difference was not statistically significant. *p < 0.0056. ABC, Autism Behaviour Checklist; ASD, autism spectrum disorder; CARS, Childhood Autism Rating Scale; PedsQL-GI, PedsQL™ 3.0 Gastrointestinal Symptoms Scales.

ABC scores before treatment and at one month and six months posttreatment showed a gradual decrease (62.26 ± 13.95, 56.79 ± 13.42, 56.42 ± 12.42) (Fig. 1A). Mauchly’s test of sphericity indicated a violation (χ^2^ = 13.68, *p* = 0.001), and Greenhouse–Geisser correction (ε = 0.644) was used. Repeated-measures ANOVA revealed a significant main time effect (F = 12.803, *p* < 0.001, partial η^2^ = 0.416, large effect). Bonferroni-corrected pairwise comparisons (α” = 0.0056) showed a significant reduction in ABC scores at only six months posttreatment relative to that before treatment (mean difference = –5.842, SE = 1.481, *p* = 0.003, 95% CI: –9.752– –1.932). By contrast, the reduction in ABC scores at one month posttreatment relative to those before treatment did not reach the corrected significance threshold (mean difference = –5.474, SE = 1.545, *p* = 0.007 > 0.0056, 95% CI: –9.552– –1.395). No significant difference was found between ABC scores at one month posttreatment and those at six months posttreatment (mean difference = –0.368, SE = 0.659, *p* > 0.0056, 95% CI: –2.107–1.370). There were differences in effect sizes across different treatment periods: before treatment vs. one month posttreatment (Cohen’s d = 0.813, large; *p *= 0.007 > 0.0056, not statistically significant), before treatment vs. six months (Cohen’s d = 0.905, large), one month posttreatment vs. six months posttreatment (Cohen’s d = 0.128, small).

#### CARS Scores

CARS scores before treatment and at one month and six months posttreatment decreased gradually (31.39 ± 4.31, 29.11 ± 3.75, 28.68 ± 3.63) (Fig. 1B). Mauchly’s test confirmed sphericity (χ^2^ = 1.010, *p* = 0.603). Repeated-measures ANOVA showed a significant main time effect (F = 36.098, *p* < 0.001, partial η^2^ = 0.667, large effect). Bonferroni-corrected pairwise comparisons (α” = 0.0056) revealed that CARS scores at one month (mean difference = –2.289, SE = 0.363, *p* < 0.001, 95% CI: –3.248– –1.300) and six months posttreatment (mean difference = –2.711, SE = 0.363, *p* < 0.001, 95% CI: –3.670– –1.752) had reduced relative to those before treatment, without a significant difference between the scores at one month posttreatment and those at six months posttreatment (mean difference = –0.421, SE = 0.299, *p* = 0.529, 95% CI: –1.211–0.369). Similarly, there were differences in effect sizes related to treatment periods: before treatment vs. one month posttreatment (Cohen’s d = 1.445, large), before treatment vs. six months posttreatment (Cohen’s d = 1.711, large), one posttreatment vs. six months posttreatment (Cohen’s d = 0.323, small).

#### PedsQL-GI Scores

PedsQL-GI scores before treatment and at one month and six months posttreatment decreased gradually (23.83 ± 13.87, 17.61 ± 10.15, 14.28 ± 8.18) (Fig. 1C). Mauchly’s test indicated a sphericity violation (χ^2^ = 21.734, *p* < 0.001), and Greenhouse–Geisser correction (ε = 0.574) was applied. Repeated-measures ANOVA showed a significant main time effect (F = 20.828, *p* < 0.001, partial η^2^ = 0.551, large effect). Bonferroni-corrected pairwise comparisons (α” = 0.0056) demonstrated that PedsQL-GI scores at one month (mean difference = –6.222, SE = 1.654, *p* = 0.005, 95% CI: –10.614– –1.831) and six months posttreatment (mean difference = –9.556, SE = 1.912, *p* < 0.001, 95% CI: –14.631– –4.480) had significantly decreased compared with those before treatment, and the scores at six months posttreatment had significantly reduced relative to those at one month posttreatment (mean difference = –3.333, SE = 0.621, *p* < 0.001, 95% CI: –4.982– –1.685). All pairwise comparisons showed large effect sizes (Cohen’s d = 0.887, 1.178, 1.265, respectively).

**Fig. 2.**
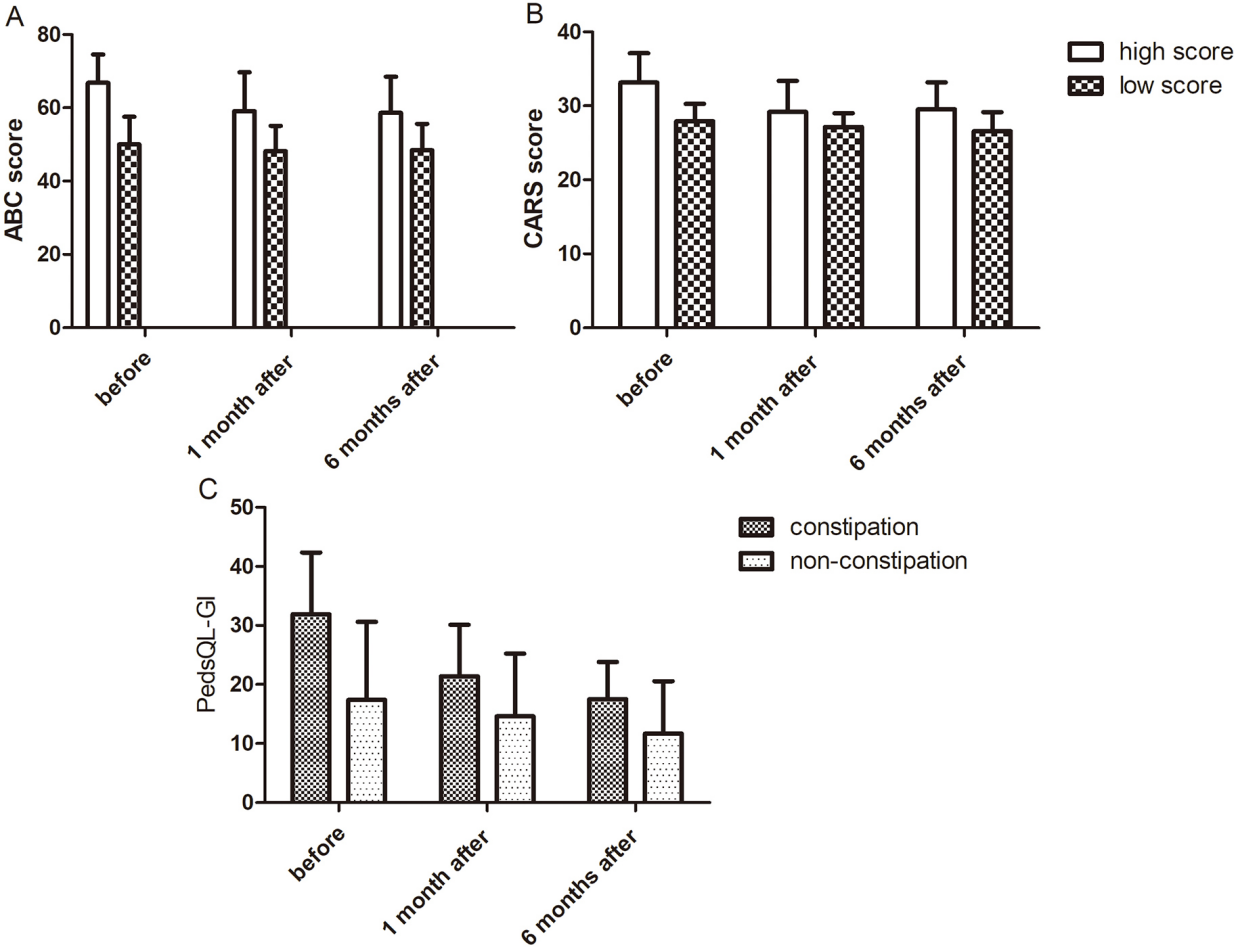
**Descriptive analysis of subgroup differences in clinical symptom scores before and after treatment (descriptive trends only, no statistical tests performed because of small subgroup sample sizes)**. (A) Comparison of ABC scores between the high- and low-score subgroups before treatment and the score reduction amplitudes after treatment. (B) Comparison of CARS scores between the high- and low-score subgroups before treatment and the score reduction amplitudes after treatment. (C) Comparison of PedsQL-GI scores between the constipation and non-constipation subgroups before treatment and the score reduction amplitudes after treatment.

As shown in Fig. 2A,B, before treatment, the ABC and CARS scores of the high-score subgroup (66.82 ± 7.76, 33.14 ± 3.98) were significantly higher than those of the low-score subgroup (50.00 ± 7.57, 27.93 ± 2.35). After treatment, the reduction amplitudes of the ABC and CARS scores of the high-score subgroup (ABC: 8.18 points, CARS: 3.95 points) were higher than those of the low-score subgroup (ABC: 1.57 points, CARS: 1.36 points). The pre-treatment PedsQL-GI score of the constipation subgroup (31.88 ± 10.49) was higher than that of the non-constipation subgroup (17.40 ± 13.20) (Fig. 2C). Posttreatment, the reduction amplitude of the PedsQL-GI score in the constipation subgroup (14.38 points) was larger than that in the non-constipation subgroup (5.70 points), whereas the reduction amplitudes of ABC and CARS scores were similar between the two subgroups. As a result of the small sample size of the subgroups, no statistical tests were performed, and only descriptive trends were presented. These results suggest that compared with other patients, those with higher baseline ABC and CARS scores had more improvements in core autism symptoms and those with more severe baseline gastrointestinal symptoms had more obvious improvements in gastrointestinal symptoms after treatment.

In addition, adverse effects, such as fever, abdominal pain, diarrhoea, vomiting and increased impulsivity or aggressiveness, were assessed after WMT. One child experienced abdominal pain after the initial WMT. However, this symptom resolved on its own within one day of the TET tube being dislodged. The remaining children did not experience any adverse effects.

### Gut Microbiota Analysis Results

**Fig. 3.**
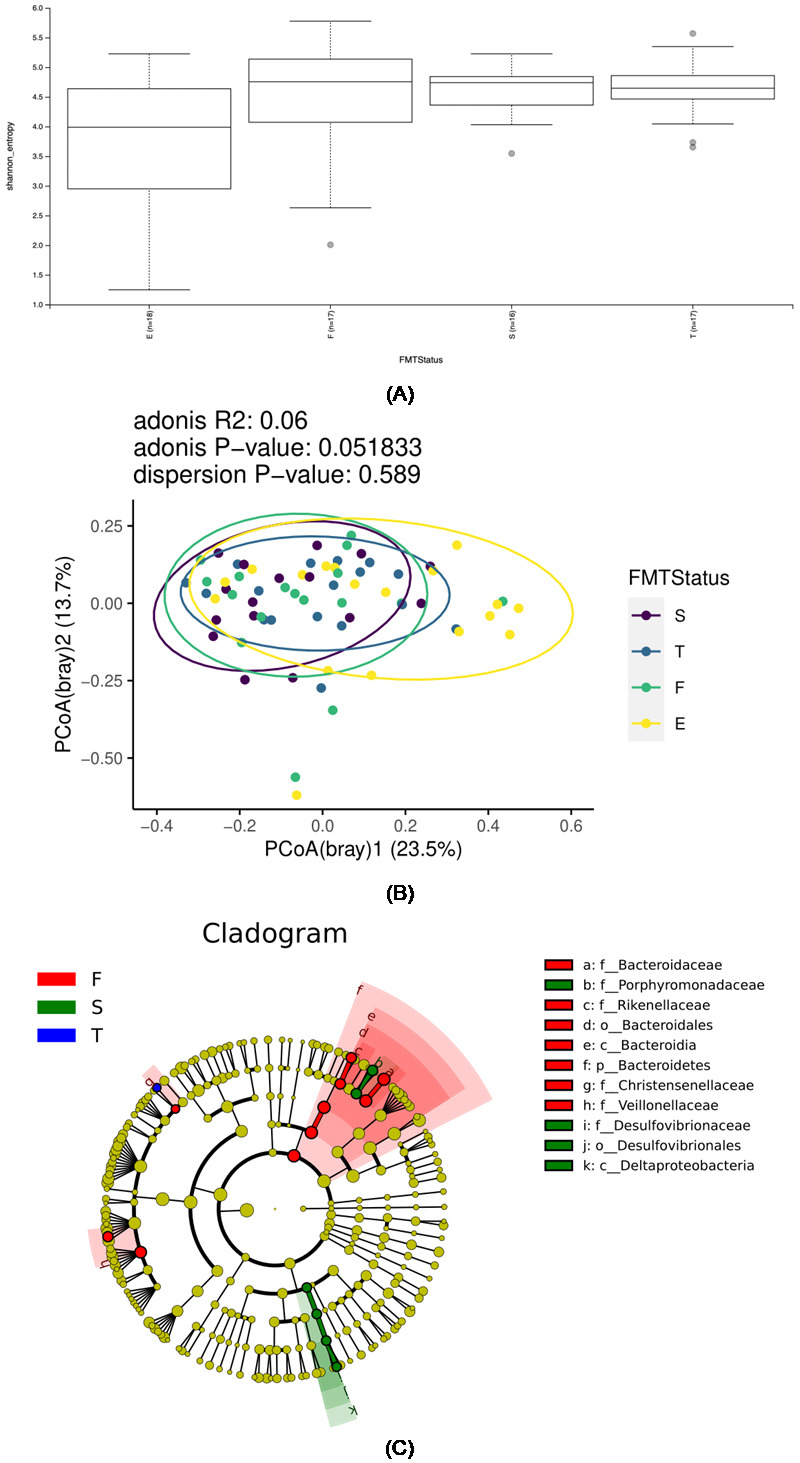
**Retrospective analysis of microbiota**. (A) Box plots of the Shannon index before and after WMT in children with typical development and those with ASD. The centreline indicates the median, and the box contains the interquartile range (25%–75%) of the data. E, normal children group; F, pre-WMT group; S, post-first-WMT group; T, post-second-WMT group. (B) PCoA of flora composition in children with ASD before and after WMT compared with that in typically developing children. (C) Evolutionary branching diagram of differential bacterial LEfSe before and after WMT in children with ASD. p_Portal level, c_Class level, o_Order level, f_Family level, g_Genus level, s_Species level. Circles radiating from the inside to the outside represent different taxonomic levels. From the inside to the outside, these levels are kingdom, phylum, order, family, genus and species. The size of the circle diameter is positively proportional to relative abundance. Yellow indicates no significant difference, red indicates a significant role in the group of normal children and green indicates a significant role in the group of children with ASD. PCoA, principal coordinate analysis; LEfSe, linear discriminant analysis effect size.

The retrospective analysis of microbiota data from 17 children who underwent two WMTs revealed that the Shannon index, an index of alpha diversity of the intestinal flora, showed an improvement in homogeneity. The 25th percentile line had elevated and the 75th percentile line had decreased after the first WMT compared with those during the pre-treatment period (Fig. 3A). Additionally, all percentile lines of the Shannon index after the second WMT remained at the same level as that after the first WMT. This finding suggests that the first WMT improved the intestinal flora, whereas the second WMT strengthened consolidation. The analysis of the alpha diversity of the intestinal flora before and after WMT showed an increase in intestinal alpha diversity in the children after the first treatment. The beta diversity of the gut microbiota was analysed through principal coordinate analysis (PCoA). PCoA (bray) 1 explained 27.65% and PCoA (bray) 2 explained 20.63% of the total structure of the intestinal flora in the normal control and ASD groups (Fig. 3B). Adonis analysis revealed a marginally significant difference in microbiota structure among all groups ($R^{2}$ = 0.06, *p* = 0.05). The centroid values and 95% confidence intervals (CIs) of each group were supplemented along the PCoA1 axis, which accounted for the largest proportion of microbiota variation, to clarify the biological importance of this marginally significant result further: the normal group (group E) had a centroid of –0.113 with a 95% CI of –0.231, 0.005; the pre-WMT group (group F) had a centroid of 0.051 with a 95% CI of –0.046, 0.148; the post-first-WMT group (group S) had a centroid of 0.115 with a 95% CI of 0.038, 0.192; and the post-second-WMT group (group T) had a centroid of 0.015 with a 95% CI of –0.076, 0.106. Notably, the 95% CIs of groups E and S showed no overlap whatsoever, a result that directly supported the marginally significant conclusion from the Adonis analysis and indicated that the difference in microbiota structures between these two groups was clearly distinguishable. The intestinal flora of children with ASD in groups E and T became similar after two WMTs, suggesting that WMT can improve the intestinal flora of children with ASD. Linear discriminant analysis effect size (LEfSe) difference analysis was used to identify the differential species at each taxonomic level to identify the differential bacteria in each group and show the differential bacteria and their abundance at each taxonomic level by using a species hierarchical relationship tree (cladogram). LEfSe difference analysis revealed that the differences in bacteria in the pre-WMT and post-first-WMT groups were different, with the predominant taxa in the pre-WMT group being Bacteroidaceae, Rikenellaceae, Bacteriodales, Bacteriodia, Bacteriodetes, Christensenellaceae and Veillonellaceae and those in the post-first-WMT groups being Porphyromonadaceae, Desulfovibrionaceae, Desulfovibrionales and Deltaproteobacteria (Fig. 3C).

**Fig. 4.**
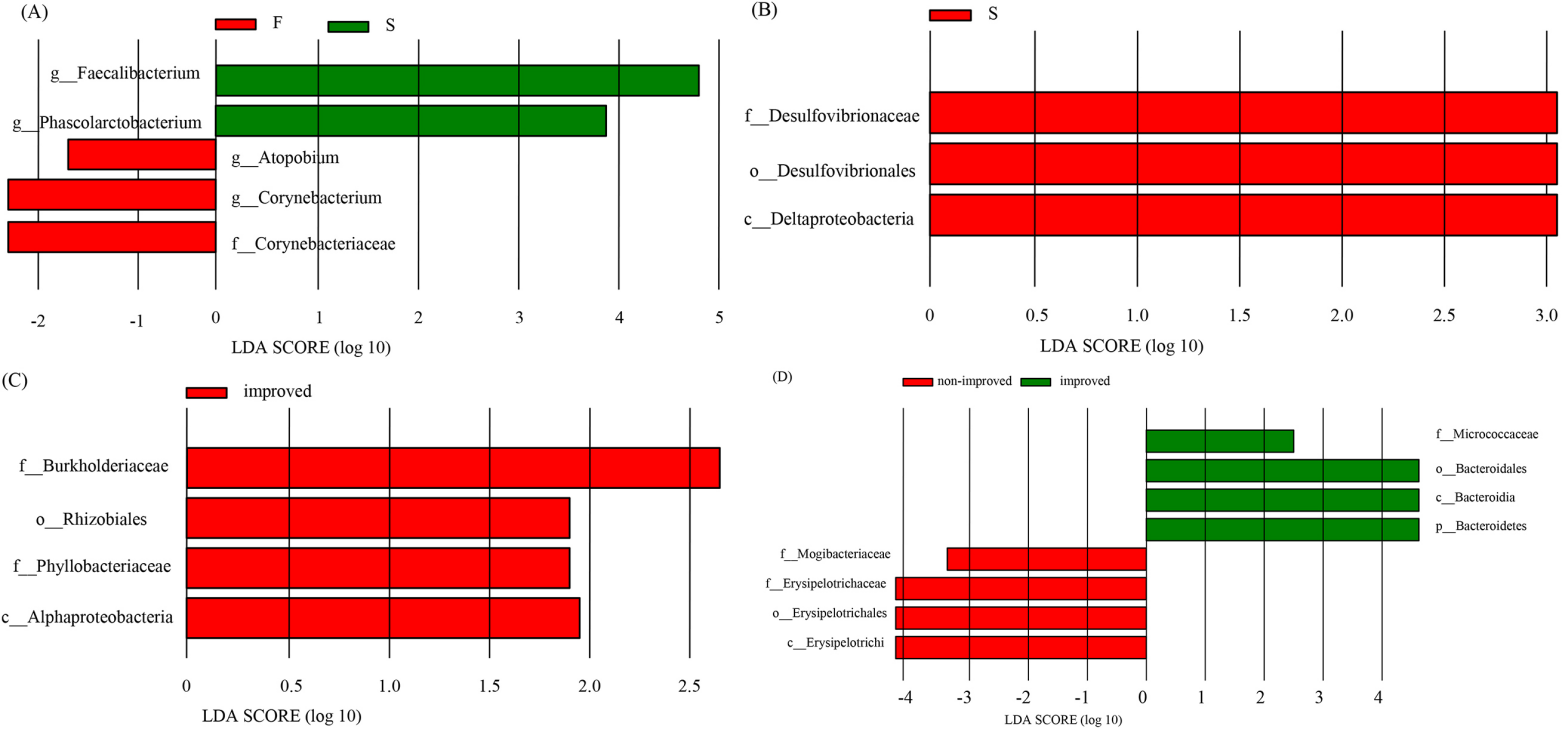
**LEfSe analysis of gut microbiota**. (A) Histogram of LDA scores for differentially abundant bacteria between F (before WMT) and S (after the first WMT) in the improvement group. (B) Histogram of LDA scores for differentially abundant bacteria at S (after the first WMT) in the non-improvement group. (C) Histogram of LDA scores for differentially abundant bacteria at F (before WMT) in the improvement group. (D) Histogram of LDA scores for differentially abundant bacteria between the improvement and non-improvement groups at S (after the first WMT). LDA, Linear Discriminant Analysis LEfSe, linear discriminant analysis effect size.

A total of 19 children with ASD were enrolled in this study and underwent follow-up assessments 12 weeks after WMT. Evaluations were jointly conducted by specialist physicians and closely accompanying family members, with treatment response defined as ABC score <67 and CGI-I score of 1–2. Amongst these patients, 12 were categorised into the improvement group (confirming sustained symptom improvement that met the aforementioned response criteria), whereas the remaining seven were assigned to the group without improvement. The intestinal flora before and after the first WMT in the groups withand without improvement were analysed by LEfSe. The length of the bar graph represents the Linear Discriminant Analysis (LDA) value, and a large value is indicative of the considerable influence of the dominant community in the group. In the improvement group, the dominant intestinal microbes after the first WMT were mainly *Faecalibacterium* and *Phascolarctobacterium* (Fig. 4A). Both of these genera are involved in the production of short-chain fatty acids (SCFAs). This finding suggests that these bacteria may be beneficial in improving children’s symptoms after WMT. In the group without improvement, the dominant intestinal microbes after the first WMT were *Desulfovibrionaceae*, *Desulfovibrionales* and *Deltaproteobacteria* (Fig. 4B). LEfSe analysis was also performed on the preoperative intestinal flora in the groups with and without improvement and revealed that before treatment, the dominant bacteria in the group with improvement were *Burkholderiaceae*, *Rhizobiales*, *Phyllobacteriaceae* and *Alphaproteobacteria* (Fig. 4C). This finding suggests that children with ASD with a high relative abundance of these microbes in their intestinal tract had better outcomes with WMT. LEfSe analysis after the first WMT in the groups with and without improvement revealed that the dominant bacteria in the group with improvement were Micrococcaceae, Bacteroidales, Bacteroidia and Bacteroidetes, whereas those in the group without improvement were *Mogibacteriaceae*, *Erysipelotrichacea*e, *Erysipelotrichales *and *Erysipelotrichi *(Fig. 4D).

## Discussion

Currently, the treatment of ASD is based on rehabilitation interventions, and no specific drug treatment for ASD exists. FMT, as an effective treatment for intestinal diseases, such as *C. difficile* infection, has shown some therapeutic efficacy in nonintestinal diseases, such as metabolic disorders and autoimmune disorders [23]. With in-depth research on the relationship between the microbe–gut–brain axis and ASD, the core symptoms and gastrointestinal symptoms in children with ASD have been found to be potentially associated with gut flora rebuilding. Therefore, the approach of improving the core symptoms of ASD by rebuilding the gut microbial environment may be a potential target for the treatment of ASD [16, 24]. A 2021 clinical study by Li *et al*. [25] found that FMT could alleviate the core symptoms of ASD; change serum neurotransmitter 5-hydroxytryptophan, γ-aminobutyric acid (GABA) and dopamine levels in children with ASD; and affect the gut flora of children with ASD to develop towards the normal paediatric gut flora.

The aim of this study is to treat children with ASD with WMT via TET placement and to evaluate the efficacy and safety of this treatment. Its findings indicate that WMT was associated with clinically meaningful improvements in ASD core symptoms (ABC and CARS) and gastrointestinal symptoms (PedsQL-GI), with significant time effects and large effect sizes (partial η^2^ = 0.416, 0.667, 0.551). Post hoc analyses clarified divergent response timelines: gastrointestinal symptoms improved rapidly and continued to advance through six months, whereas core autism symptoms required a long intervention period (with significant reductions in ABC occurring only at six months posttreatment) and stabilised after initial improvement. Descriptive subgroup analyses further suggested that WMT may be more beneficial for children with severe baseline core symptoms (high ABC/CARS scores) and prominent baseline gastrointestinal symptoms (constipation subgroup) than for other children. These data support the potential efficacy of WMT in alleviating the core and gastrointestinal symptoms of ASD and highlight that baseline symptom severity may be a key factor modulating treatment response, an important consideration for personalised WMT intervention strategies. This consideration warrants further validation in large cohorts. In this study, the WMT technique was used, and the safety of the treatment was higher than that of traditional FMT. During the treatment period, only one child had a transient abdominal pain symptom after the first WMT. This symptom resolved on its own after the TET tube was dislodged for one day, proving that the WMT treatment of ASD in children is very safe. At present, domestic FMT is mainly performed via oral, enema, or colonoscopic treatment strategies, and TET tube placement has been found to be better than enema [26]. TET tube placement can be used several times for deep intestinal WMT after tube placement such that the washed bacterial fluid of the donor can be widely distributed throughout the whole colon to facilitate improved colonisation.

Previous studies have reported that the intestinal flora of patients with ASD is remarkably different from that of healthy controls [27], suggesting that intestinal flora dysbiosis may be associated with the development of ASD. The analysis of bacterial flora results in this study also found that the intestinal flora of children with ASD differed from that of normal children, and WMT can improve the intestinal flora and increase beneficial bacteria in children with ASD. In the group with improvement, the dominant strains changed to *Faecalibacterium* and *Phascolarctobacterium*, which are also involved in the production of intestinal SCFAs [28, 29]. Notably, the present study found that the pre-treatment intestinal differential bacteria in the group with improvement were mainly *Proteus*, which was not observed in the group without improvement. Previous study has reported that *Proteus*is elevated in various intestinal disorders, such as inflammatory bowel disease, colorectal cancer, necrotising small intestinal colitis and irritable bowel syndrome, and the large number of *Proteus* in the intestines can reflect imbalance in the intestinal flora [30]. This finding suggests that the degree of intestinal dysbiosis may be a potential predictor of WMT response in children with ASD. Specifically, children with a high abundance of *Proteus* before treatment may experience a better therapeutic effect than those without. This observation provides a novel direction for optimising WMT strategies because pre-treatment intestinal flora analysis could help identify suitable candidates and improve treatment efficiency.

After the first WMT treatment, the intestinal differential bacteria in the group with improvement were mainly members of Bacteroides, which was not detected in the group without improvement. The present study’s focus on posttreatment Bacteroides enrichment aligns with previous reports showing that this genus participates in the production of the serum neurotransmitter GABA [31], a molecule closely related to cognitive function. This finding supports a potential association between WMT-induced changes in intestinal flora and the improvement of core symptoms in children with ASD because the enrichment of Bacteroides may promote GABA production. This effect may, in turn, be associated with the modulation of cognitive function. The regulatory effect of Bacteroides and Faecalibacterium, the dominant strains in the group with improvement, on ASD symptoms may be associated with the modulation of ASD symptoms via the brain–gut axis, with SCFAs and GABA serving as key signalling molecules. Specifically, Faecalibacterium is a major producer of butyrate (a type of SCFA), whereas Bacteroides can synthesise acetate and propionate. These SCFAs derived from the two key strains may cross the blood–brain barrier and be associated with the regulation of central nervous system (CNS) neuron activity by inhibiting histone deacetylases [32, 33, 34], which could be linked to improvements in cognitive deficits and emotional disorders in ASD. Additionally, Bacteroides participates in the synthesis of GABA, a major inhibitory neurotransmitter in the CNS. Through the brain–gut axis, GABA produced by Bacteroides may be transported to the CNS via the circulatory system, which could be associated with the regulation of neural circuit excitatory–inhibitory balance and the potential alleviation of the core behavioural symptoms of ASD (e.g., social communication deficits and repetitive behaviours) [35]. This pathway analysis is consistent with our study’s observation that the enrichment of Bacteroides and Faecalibacterium in the group with improvement is associated with symptom amelioration, supporting a potential biological mechanism that may link gut microbiota shifts to ASD symptom improvement.

Additionally, the present study identified Bacteroides and F. prausnitzii as key strains in the group with improvement. These strains are known to be functional constituents in FMT. Previous studies have demonstrated that these two strains act synergistically: Bacteroides consumes oxygen to facilitate colonisation by the strictly anaerobic F. prausnitzii and provides metabolic precursors for butyrate synthesis [31]. The present findings support the potential of these strains as core candidates for targeted microbial therapy in ASD, moving beyond the heterogeneity of conventional FMT. Therefore, this study underscores the value of WMT in reshaping the intestinal flora of children with ASD and identifies specific strains associated with treatment response. Its findings provide a foundation for developing precision microbial therapies for ASD based on the distinct strains identified in this study. Given the limitations of the present study’s design, SCFAs, inflammatory markers, or neurotransmitter levels were not detected. Future studies are recommended to combine intestinal flora analysis with the detection of the above indicators to verify the mechanism of WMT in children with ASD further.

The emerging application of artificial intelligence in characterising the gut microbiome of individuals with ASD presents a promising direction for personalising therapeutic interventions [36]. The authors’ own investigations, which identified specific bacterial strains associated with treatment response through the longitudinal analysis of faecal microbiota transplantation, contribute data that could support this development. Future work focusing on the integration of such microbial features into predictive models may help stratify patients prior to treatment. This approach could guide the selection of candidates for microbiota-based therapies, like FMT, thereby potentially improving their precision and clinical success.

This study has several limitations that should be acknowledged. Firstly, its retrospective design may introduce potential selection bias, which restricts the generalisability of its results. Secondly, although the medication dose was calculated on the basis of age and body weight, age may independently affect the therapeutic effect through differences in physiological development, and the failure to conduct stratified analysis on the independent role of age further may affect the interpretation of the results. Thirdly, although the difference in intervention frequency was caused by the objective uncontrollable factor of the COVID-19 pandemic, it may still introduce systematic bias, which could have a potential effect on the evaluation of intervention effects. Future studies should expand the sample size, adopt a prospective design, employ stratified analysis to control the independent effect of age and reduce the effect of external factors on intervention consistency through optimised study designs.

## Conclusions

This preliminary retrospective analysis suggests that WMT may potentially alleviate gastrointestinal and core behavioural symptoms in children with ASD, with more pronounced improvements in constipated individuals and those within the high-severity subgroup than in other individuals. However, the small sample size (n = 19), borderline or nonsignificant p values for key outcomes, retrospective design and external factor interference restrict the generalisability of this study’s findings, which require cautious interpretation. Large, rigorous prospective studies are warranted to validate this study’s observations and clarify the therapeutic potential of WMT for paediatric ASD.

Critically, *Faecalibacterium* and *Phascolarctobacterium* were enriched in patients with clinical improvement. These microbial signatures provide preliminary clues for exploring the potential mechanism of WMT and represent candidate biomarkers for predicting treatment response. This finding provides a crucial clue for advancing towards a potential personalised, microbiota-targeted therapy for ASD, where patient stratification might be guided by individual gut microbiome profiles.

The lack of standardised assessment tools may affect cross-study comparisons. In addition, further research using controlled experimental designs is needed to investigate the specific causal mechanisms by which microbiota transplantation improves therapeutic effects in children with autism spectrum disorder.

## Availability of Data and Materials

All experimental data included in this study can be obtained by contacting the corresponding author if needed.
